# Timing of human rhythmic jumping with vertical floor vibrations tends towards mechanically efficient solutions

**DOI:** 10.1038/s41598-025-92998-3

**Published:** 2025-03-15

**Authors:** Nimmy Mariam Abraham, Stana Zivanovic, Genevieve Williams

**Affiliations:** 1 Faculty of Environment, Science and Economy, University of Exeter, Exeter, Devon, EX4 4QF UK; 2 School of Engineering, University of Warwick, Coventry, West Midlands, CV4 7AL UK; 3 Faculty of Health and Life Sciences, University of Exeter, Exeter, Devon, EX2 4TA UK

**Keywords:** Rhythmic jumping, Vibrations, Jump timing, Biomechanics, Human-structure interaction, Civil engineering, Biomechanics

## Abstract

Jumping on vibrating platforms is described not only by the frequency of jumping (FoJ) but also by the timing of key events in a cycle of jumping relative to vibrations. This study aimed to capture timing and efficiency-related adaptations during jumping on vertically vibrating platforms. Whole body kinematic and kinetic data were collected as ten participants jumped on a sinusoidally vibrating platform of 2.0, 2.4 and 2.8 Hz at 2 m/s^2^. FoJ matched platform frequency, and audio cues were provided to time the jump landing at four positions relative to the platform position: reference position on its way down, lowest position, reference position on its way up, and highest position. FoJ, jump timing, impact factor, contact ratio, mechanical work and leg stiffness were calculated for each jump cycle. Results confirmed that the impact factor, contact ratio, mechanical work, and leg stiffness are timing-dependent. Results also showed that despite being cued, participants adjusted their timing to take off from a higher platform position during its downward motion and land at a lower position during the upward motion while maintaining FoJ. Importantly, participants tended towards efficiency by employing jump timings related to lower energy input, appropriate contact ratio and lower forces. This study provides evidence of jump timing behaviour relative to platform motion being dominated by the efficiency of jumping. Practically, it may be crucial to consider this aspect when estimating human-induced loads on lively assembly structures.

## Introduction

Contemporary sustainable structures like grandstands, footbridges, and floors are lively and susceptible to human-induced dynamic excitation due to rhythmic jumping, for example during sporting events, music concerts or civic and religious events. Rhythmic jumping can potentially initiate large structural vibrations that pose a significant risk to spectators’ comfort and occasionally to structural safety. The vibrations could force a rhythmic jumper to adjust their body movement to the structural motion, altering the previously expected structural vibration response and the properties of the dynamic system. These mutual and continuously evolving influences of the structure on the human and human on the structure are collectively referred to as human-structure interaction. Mechanical efficiency, defined as the ratio of useful mechanical work output to the total energy input, may influence human adaptations. The work output is the total mechanical work done between the human body’s centre-of-mass (CoM) and platform motion, whereas the energy input is the human effort to sustain rhythmic jumping. Maintaining a constant jump height, humans might adjust their jumping to harness energy from vibrations to sustain rhythmic jumping reducing their efforts and avoiding injury risks. This study investigates the effects of vertical vibrations on in-place bilateral rhythmic jumping to understand its potential impact on structures.

**Fig. 1:**
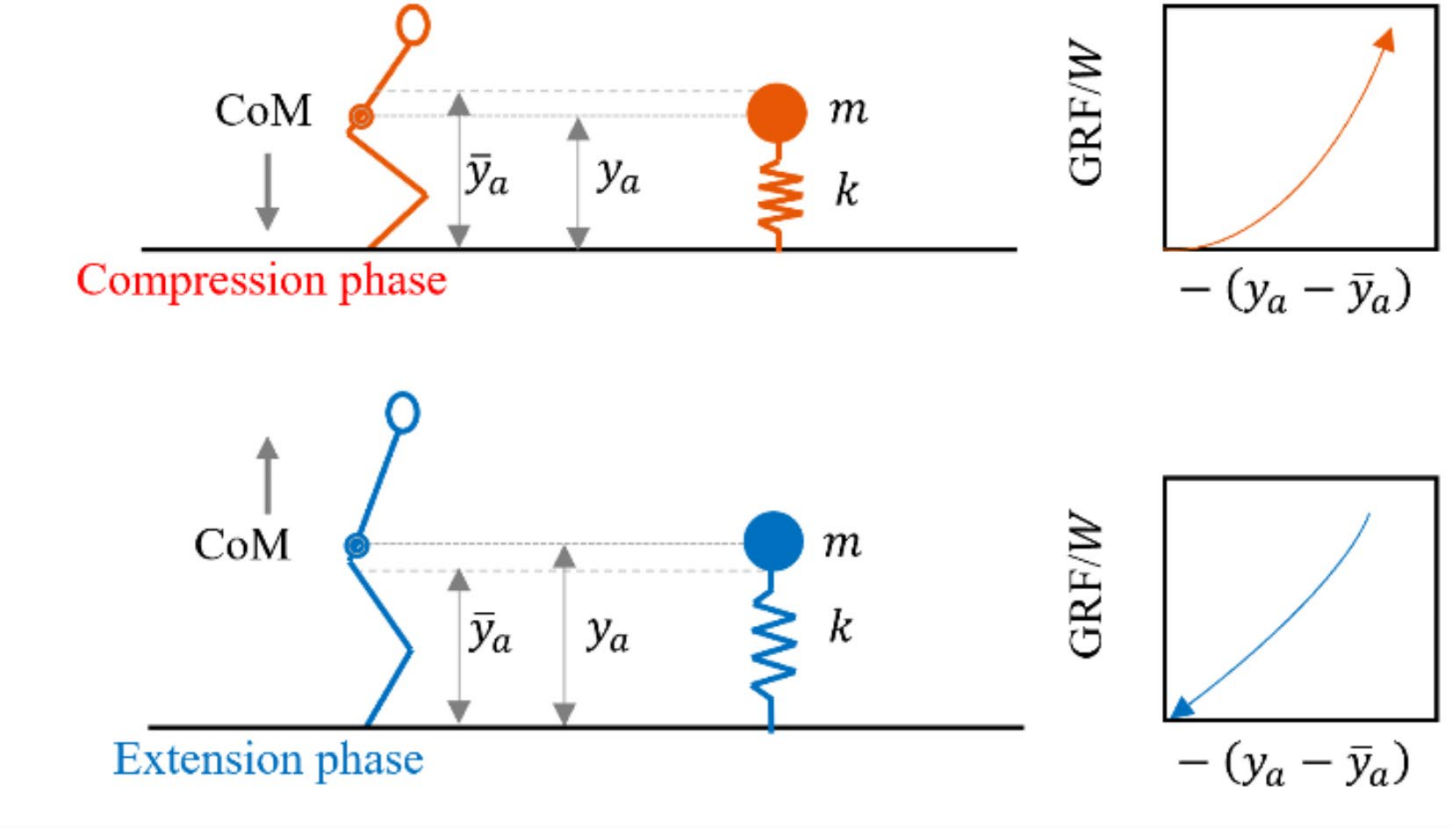
Illustration of a jumping human as a linear spring-mass model showing the body centre-of-mass (CoM)’s vertical motion, spring-mass idealisation and the relationship between generated vertical ground reaction force (GRF) and CoM displacement. ${y}_{a}$ is the CoM position, ${\overline{y} }_{a}$ is the mean CoM position, $W$ is the body weight of the jumper. Sign convention: GRF is positive downwards and the CoM’s displacement from its mean position, (${y}_{a} - {\overline{y} }_{a}$), is positive upwards. A jumping person is approximated as a point mass bouncing on a massless spring without viscous losses^1,4,8^. The point mass, $m$, represents the body mass and the spring constant, $k$, represents the leg stiffness (i.e., GRF generated per unit CoM displacement) of the person. The contact phase in a cycle of jumping where GRFs exist comprises compression and extension phases^9^. Compression occurs during the body CoM’s downward motion, engaging the muscles to absorb and control the impact forces generated. This phase is characterised by flexion at the hip, knee, and ankle joints. GRF typically increases from zero at landing until it reaches its peak at about the CoM’s lowest position in the contact phase (LCP). Extension that follows compression involves an upward movement of the CoM. The stored energy from the compression is released in this phase, extending the hip, knee, and ankle joints, pushing against the supporting platform to generate vertical lift^1,10,11^. The GRF decreases to zero at the take-off and remains zero in the flight phase. The CoM displacement at its highest position in the flight phase (HFP) positively correlates with the generated GRF^3^. The area under the force–displacement curve represents the mechanical work and the mean slope of the curve indicates leg stiffness during each phase. The work done during compression is positive while it is negative during extension.

Bilateral rhythmic jumping is primarily characterised by a repeated alternation between contact and flight phases at a given frequency^1–3^. It is physiologically possible in a narrow frequency band of 1–4 Hz^3–7^, the limits defined by muscle properties and skeletal dimensions^1^. The preferred or most comfortable jumping is between 2.0 and 2.8 Hz^2,5^, where the energy storage and release is the largest^1,4^. Prior studies established that humans choose the frequency at which they behave most like spring-mass systems (Fig. 1) to benefit power output optimisation and mechanical cost reduction. They adjust their leg stiffness to accommodate the variations in mechanical demands due to changes in the frequency of jumping (FoJ)^4,12–14^. While legs act like springs in the preferred FoJ range, they cease to do so at lower FoJs due to separate heel and toe actions, i.e., the toe makes initial contact with the ground followed by the heel. This is indicated by a double peak in the time history of ground reaction force (GRF) within a single cycle of jumping^15–17^. Metabolic cost was found to be higher at lower FoJs^18^. On the other hand, at higher FoJs, although the spring-like behaviour is maintained, the lower contact ratio (i.e., the ratio of the contact duration to the period of the cycle of jumping) results in an efficiency reduction due to faster generation of muscular force^4^. Legs behave like a strut that produces high force with minimal displacements^12^. At the preferred frequency range, it is the spring-like behaviour and the optimal contact ratio that maximise the efficiency of jumping^4^.

Further studies that explored the effect of surface conditions revealed that humans jumping on elastic platforms are spring-like systems in series^19–21^. Elastic surfaces also store and release energy which humans harness by adjusting their leg stiffness to reduce the muscular work required to sustain jumping. Thus, a constant combined stiffness of the system is maintained^19–21^. Conversely, energy gets dissipated on damped surfaces where humans perform additional muscular work behaving like a work-producing actuator to sustain rhythmic jumping^22,23^. This adjustment results in greater energy release during the extension phase than stored during the compression phase^22^. Indeed, understanding these behaviours can help inform aspects of human-structure interaction and even the design of sports shoes and exoskeletons^23^.

The dynamics of rhythmic jumping on vibrating platforms are complex compared to stationary platforms. They include not only the FoJ but also the timing of key events in a cycle of jumping relative to the events in the vibration cycle^24^. The few studies in the literature that examined rhythmic jumping on vibrating surfaces observed the impossibility of jumping in synchronisation with large vibrations, and the reduction in GRFs when attempting to do so^5,25^. The modal accelerations in those studies were between 1.5 and 2.2 $g$, where $g=$ 9.81 m/s^2^ is the acceleration due to gravity. Although those studies presented crucial experimental observations, the authors investigated neither the causes nor the specific vibration levels that affected jumping. Another observation was the increase in human leg stiffness with an increase in the FoJ similar to that on the stationary platforms^9^. The timing of jumps was not considered in experiments and subsequent analyses in prior studies. The authors’ recent work^24,26^, however, found that the GRFs are dependent on the timing and that jumpers adjust their timing while jumping on vibrating platforms which results in deviating from the audio cues. Thus, the relationship between jump timing and mechanical efficiency merits further research.

This study focuses on the effect of vertical vibrations on in-place bilateral rhythmic jumping. It is hypothesised that humans adapt their jumping relative to floor vibrations towards efficiency, i.e., maintaining jump height and therefore without additional energy input related to CoM motion, harnessing energy from platform motion. The aim is to understand efficiency-dependent behaviours related to cued jump timing on vibrating platforms as well as their biomechanical underpinning by utilising experimental observations and physical interpretations. To address this aim, biomechanical characteristics of rhythmic jumping on a vibrating platform were compared with those during jumping on a stationary platform. The following jump parameters were analysed: FoJ, jump timing, impact factor (i.e., the ratio of peak GRF to the participant’s body weight^15–17^), jump height (i.e., the peak displacement of centre-of-mass, CoM, from its mean position), contact ratio, mechanical work (total work done between the CoM and platform motion in scenarios with platform motion and work produced due to the CoM motion in scenarios without platform motion), and leg stiffness. The behaviours relating to jumping at low (2.0 Hz), moderate (2.4 Hz) and high (2.8 Hz) FoJs where metronome beats prompted the target landing instance of each cycle were studied. Four timings used in this study were landing at the platform’s reference position on its way down or mid-down (A), lowest position or trough (B), reference position on its way up or mid-up (C), and highest position or peak (D)^24^.

## Results

### Jumping on the stationary platform

**Fig. 2:**
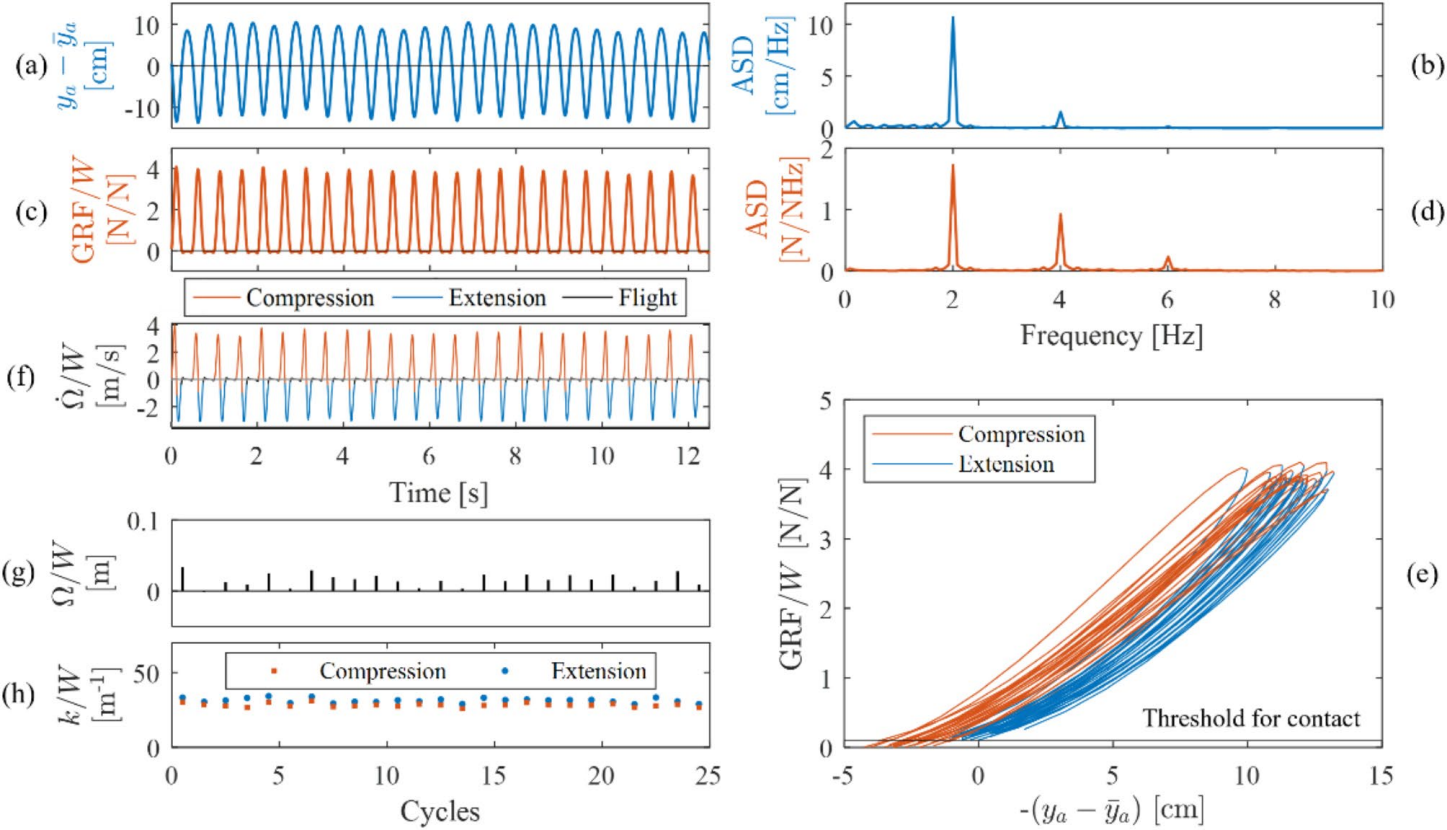
Example showing the (**a**) time history and (**b**) Fourier spectrum (depicting amplitude spectral density, ASD) of CoM displacement with mean subtracted, (${y}_{a} - {\overline{y} }_{a}$), (**c**) time history and (**d**) Fourier spectrum (depicting ASD) of GRF, (**e**) force–displacement relationship during the contact phase, (**f**) instantaneous power or rate of work done, $\dot{\Omega }$, (**g**) cycle-by-cycle work done, $\Omega$, and (**h**) cycle-by-cycle stiffness, $k$, corresponding to the compression and extension phases of the fourth participant’s third trial of jumping at 2.0 Hz on the stationary platform. Quantities on the y-axis in (**c**–**h**) are normalised by the participant’s weight, $W$. The Fourier spectrum in (**d**) does not include the static component. The area under the curve in (**e**) is the work done during the respective phases. The net work done is the difference between the area corresponding to the two phases. The time history of instantaneous power obtained as the product of GRF and velocity (rate of displacement) is plotted in (**f**). Positive power denotes compression and negative power indicates extension. The area under the power curve of each cycle in (**f**) is plotted as cycle-by-cycle work in (**g**)^27^. When work done during the compression phase is greater than during the extension phase of a cycle, the net work done is positive and vice versa. A net positive work indicates that the energy generated during landing exceeds the energy released to take off. In contrast, a net negative work shows that the energy generated during landing is lower than the energy released to take off. Additionally, the mean slope of the force–displacement curve in (**e**) indicates the leg stiffness^9^.

A representative example of time histories of CoM displacement and GRF, force–displacement relationship, instantaneous power, cycle-by-cycle mechanical work and cycle-by-cycle leg stiffness for audio-cued jumping at 2 Hz on the stationary platform is shown in Fig. 2. Consistency is observed in the peaks of both CoM displacement (Fig. 2a) and GRF (Fig. 2c) across the cycles, except for the inherent cycle-by-cycle variability. This variability in time history manifests as an energy spread around the strong harmonics in the Fourier spectrum (Fig. 2b,d). The participants achieved frequencies within ± 0.1 Hz of target FoJ in 77, 78 and 65% of the cycles of jumping with 2.0, 2.4 and 2.8 Hz targets, respectively. Mechanical work was positive and consistent across the cycles except for the inherent cycle-by-cycle variability (Fig. 2g). The cycle-by-cycle stiffness during extension was slightly greater than compression (Fig. 2h).

**Fig. 3:**
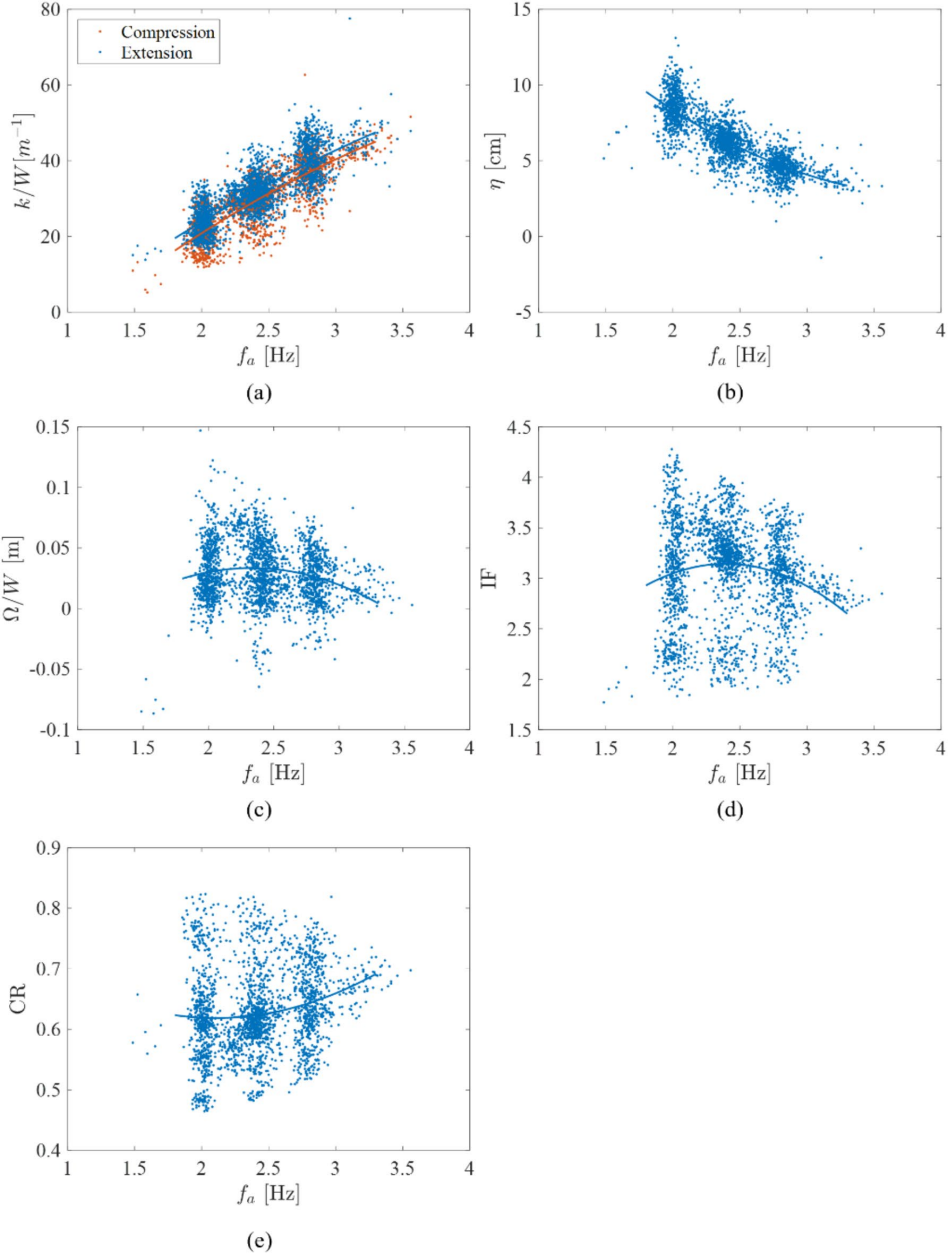
Effect of FoJ, ${f}_{a}$, on (**a**) normalised stiffness, $k/W$, (**b**) jump height, $\eta$ (**c**) normalised work, $\Omega /W$, (**d**) impact factor, IF, and (**e**) contact ratio, CR; The cycle-by-cycle values on the stationary platform are plotted and quadratic fits are shown.

The cycle-by-cycle stiffness was positively correlated with FoJ (Fig. 3a) whereas the cycle-by-cycle jump height was negatively correlated with FoJ (Fig. 3b). The mean jump heights were 8.23, 6.15 and 4.17 cm at 2.0, 2.4 and 2.8 Hz, respectively (Fig. 3b). Work and impact factor were the greatest near FoJ of 2.4 Hz (Fig. 3c,d) while the contact ratio was the lowest near FoJ of 2.4 Hz (Fig. 3e).

### Jumping on the vibrating platform

The vibration amplitude of 2 m/s^2^ used in the tests yields peak platform displacements of 1.27, 0.88 and 0.65 cm at the vibration frequencies of 2.0, 2.4 and 2.8 Hz, respectively. The participants were asked to jump at audio-cued frequencies that matched the vibration frequency. The cues were set at four timings: A, B, C and D at each FoJ. The achieved cycle-by-cycle jump timings were calculated as angular values in the range [0°, 360°) based on the relative phase between the displacements of CoM and the platform. The four timings, when executed to perfection (i.e., adhering to metronome beats), correspond to relative phase values of 0°, 90°, 180° and 270°, respectively. Furthermore, jumping consistently at the same frequency as platform vibrations results in consistent jump timing relative to vibrations across the cycles.

**Fig. 4:**
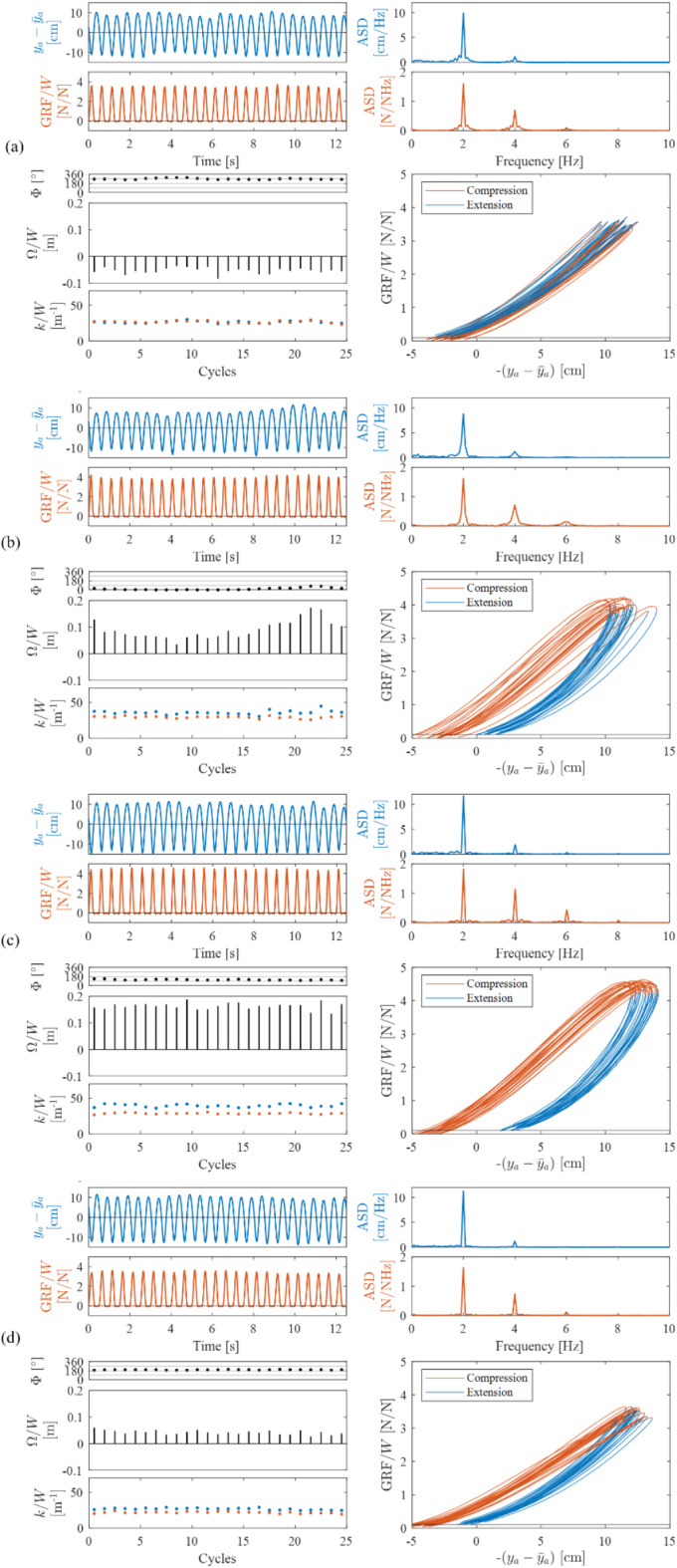
Examples showing the profiles of the time history and Fourier spectrum (depicting ASD) of CoM displacement with mean subtracted, (${y}_{a} - {\overline{y} }_{a}$), and time-history and Fourier spectrum (depicting ASD) of normalised GRF, GRF $/W$, cycle-by-cycle timing, $\Phi$, force–displacement relationship, normalised cycle-by-cycle work done, $\Omega /W$, and normalised cycle-by-cycle stiffness, $k/W$, corresponding to compression and extension phases for the fourth participant’s third trial of jumping at 2.0 Hz with target timings: (**a**) A, (**b**) B, (**c**) C and (**d**) D. The achieved timings were closer to timing values of 270°, 0°, 90°, and 180° for targets 0°, 90°, 180°, and 270° (**a**–**d**), respectively. The Fourier spectrum does not include the static component.

The participants achieved frequencies within ± 0.1 Hz of target FoJ in 79, 79 and 72% of the cycles of jumping with 2.0, 2.4 and 2.8 Hz targets, respectively. Time histories of CoM displacement and GRF, force–displacement relationship, cycle-by-cycle jump timing, cycle-by-cycle mechanical work and cycle-by-cycle leg stiffness for exemplary trials of jumping at four target timings at 2.0 Hz on the vibrating platform are shown in Fig. 4. Jumping at a target of 2.0 Hz FoJ in Fig. 4a–d shows that the achieved timings were consistent throughout. However, the actual relative phase values were closer to 270°, 0°, 90°, and 180° than the targets of 0°, 90°, 180°, and 270°, respectively. This implies that the participant in this scenario achieved nearly consistent timing but was a quarter cycle early at all four target timings at 2.0 Hz.

In Fig. 4a, where the achieved relative phase values were close to 270°, there was a destructive interference between the kinematics of the participant’s CoM and platform, resulting in lower peak GRFs than on the benchmark stationary platform (Fig. 2c). The cycle-by-cycle net mechanical work was negative across the cycles of jumping implying that the energy spent for take-off was greater than the energy generated during landing. In this scenario, the leg stiffness (Fig. 4a) was lower than for jumping on the stationary platform (Fig. 2h) and the legs were stiffer during compression than extension. Conversely, constructive interference was observed when the achieved relative phase was near 90° (Fig. 4c), which resulted in peak GRFs greater than on the stationary platform (Fig. 2c). The cycle-by-cycle net positive work was the greatest among the scenarios of jumping on the stationary (Fig. 2g) and vibrating platforms (Fig. 4). This implies that the energy generated during landing exceeded the energy spent for take-off. Here, the leg stiffness was also the greatest among the scenarios of jumping on the stationary platform (Fig. 2h) and vibrating platforms (Fig. 4). The legs were stiffer during extension than compression with hysteresis in the force–displacement curve (Fig. 4c), thus behaving differently from an ideal spring-mass system. For jumping whereby the achieved relative phase was 0° (Fig. 4b) and 180° (Fig. 4d), the CoM kinematics, GRFs, work and leg stiffness were similar to jumping on the stationary platform (Fig. 2) with no interference. The near-consistent jump timing across the cycles of each trial resulted in near-consistent peak CoM displacements, peak GRFs, work and stiffness across the cycles of trials at all four target timings (Fig. 4). The slight inconsistency in timing in the latter half of the trial in Fig. 4b resulted in corresponding inconsistent peak CoM displacements, GRFs, work and stiffness values.

**Fig. 5:**
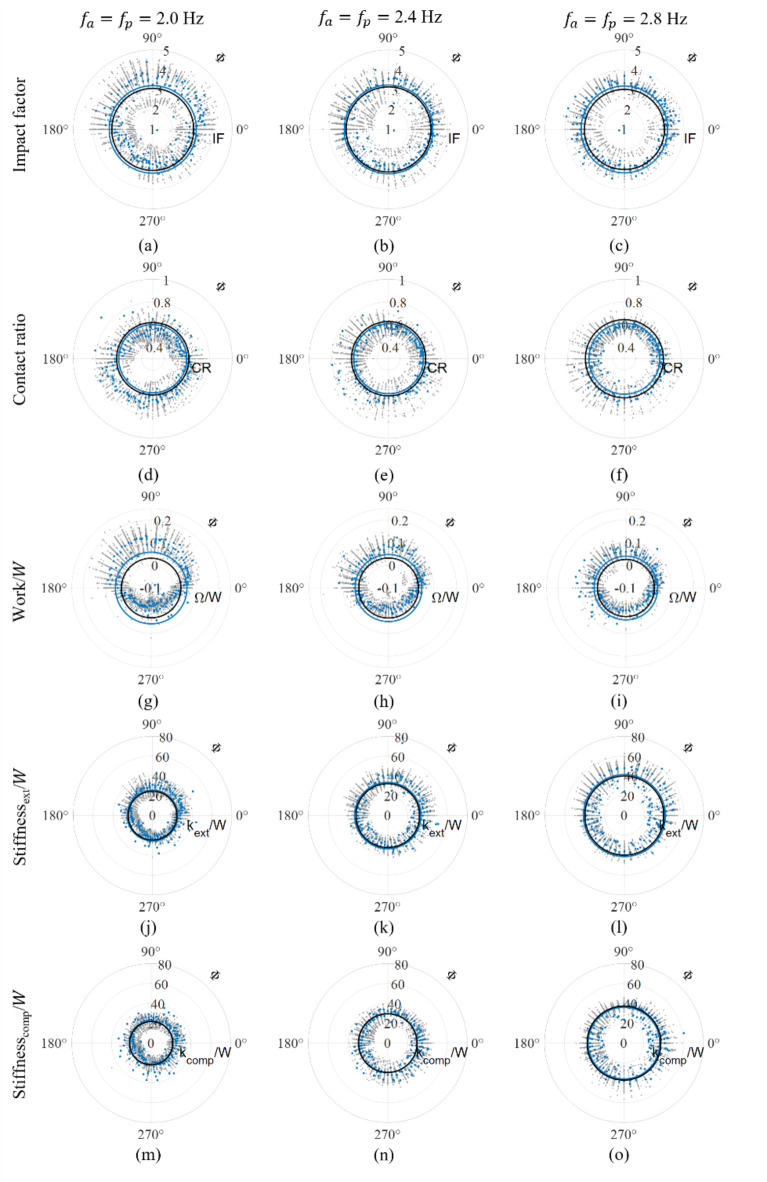
Variation of cycle-by-cycle impact factor, IF, contact ratio, CR, normalised work done, $\Omega /W$, normalised stiffness during extension, ${k}_{ext}/W$, and normalised stiffness during compression, ${k}_{comp}/W$, across the range of timing, $\Phi$, by all participants for the FoJs: 2.0 Hz, 2.4 Hz and 2.8 Hz; ${f}_{a}$ is FoJ and ${f}_{p}$ is vibration frequency; The mean at each timing value is indicated using blue markers; The overall mean is represented using a blue circle; The mean for corresponding FoJ on the stationary platform is indicated using a black circle.

The effect of jump timing on cycle-by-cycle jump parameters is visualised for each FoJ in Fig. 5. The overall mean values of the cycle-by-cycle impact factor (Fig. 5a–c), contact ratio (Fig. 5d–f), net mechanical work (Fig. 5g–i) and leg stiffness (Fig. 5j–o) at each target FoJ on the vibrating platform were similar to those on the stationary platform. However, impact factor, work and stiffness were greater for the cycles of jumping with a relative phase value between 0° and 180° and lower for those with a relative phase value between 180° and 360° compared to the corresponding overall mean values. The trend is the opposite for the contact ratio. Moreover, the mean values of these jump parameters at relative phase values near 0° and 180° approached the overall mean (Fig. 5). Additionally, the mean cycle-by-cycle stiffness increased as the FoJ increased (Fig. 5j–o).

**Fig. 6:**
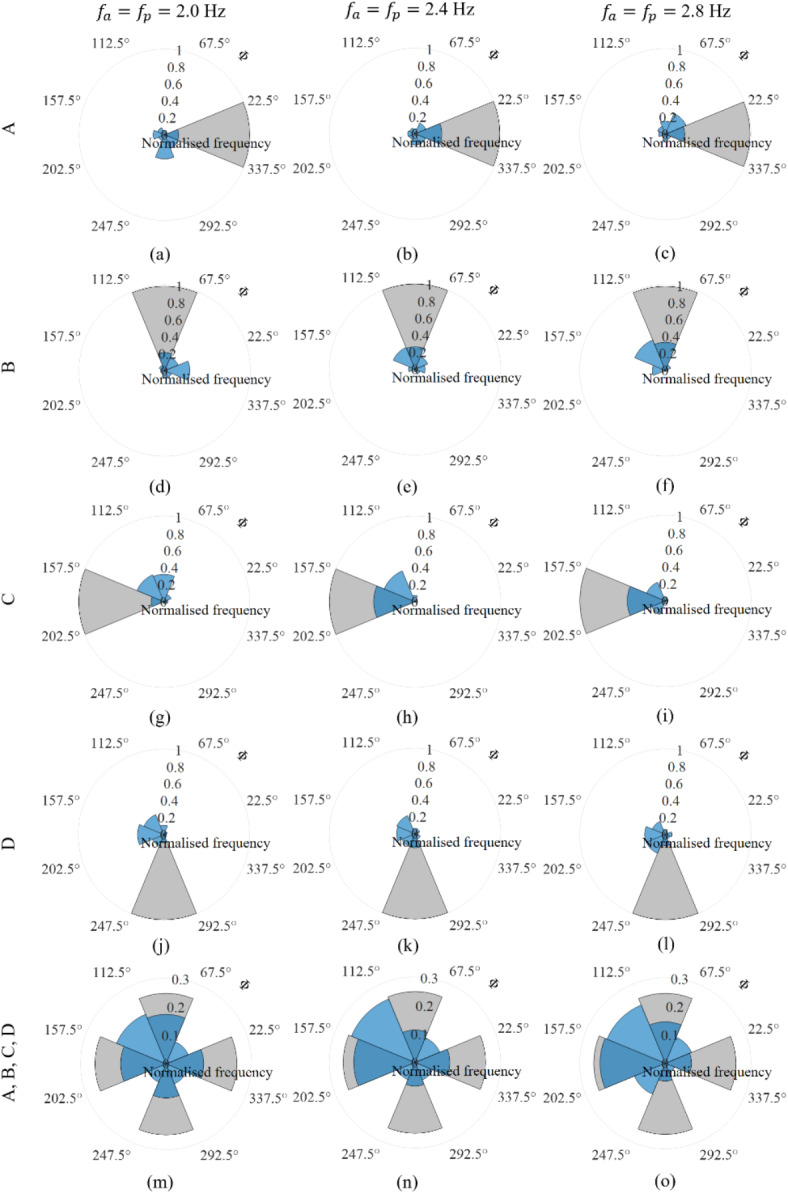
Distribution of cycle-by-cycle timing, $\Phi$, by all participants (blue) and the respective targets (grey). Polar histograms are presented separately for the 12 configurations in (**a**–**l**) and together at each target FoJ in (**m**–**o**). The cycle-by-cycle timing values were divided between eight bins for each case to understand its distribution. Each bin was 45° wide, with boundaries set at 22.5°, 67.5°, 112.5°, 157.5°, 202.5°, 247.5°, 292.5°and 337.5°. The normalised frequency along the radial axis represents the proportion of jump cycles within a specific timing range or bin, calculated by dividing the number of jumps in that bin by the total number of jumps in the histogram. If the participants executed jumping at perfect timing in all the tests, the bins 337.5–22.5°, 67.5–112.5°, 157.5–202.5°, and 247.5–292.5° would have 100% of the data points in (**a**–**l**), respectively, and 25% of the data points each in each of these four bins (target ± 22.5°) in (**m**–**o**), as indicated using grey shade. Allowing for slight variations in the timing, these four bins are still expected to dominate whilst a small amount of the data would fall in the neighbouring bins (target ± 67.5°). However, in (**m**–**o**) there is a dominance in bins of 135° ± 67.5° against the expectation. The bin encompassing 315° is the least concentrated.

Figure 6 depicts the distribution of jump timing in comparison with the respective targets using polar histograms. The cycle-by-cycle timing values were divided between eight bins each 45° wide (target ± 22.5°). If the participants executed jumping at perfect timing in all the tests, the target bins would have 100% of the data points in Fig. 6a–l, where histograms are plotted separately for each target timing and FoJ. However, the distribution for jumping at 2.0 Hz showed a general tendency to jump slightly earlier or on target (Fig. 6a,d,g,j,m). Few jumps had a relative phase of around 135° at all four target timings (Fig. 6a,d,g,j). At 2.4 Hz (Fig. 6b,e,h,k,n), the tendency was to jump either precisely at the target, except when the target was 270° (Fig. 6k), or incline towards the 135° relative phase, except when the target was 0° (Fig. 6b). At 2.8 Hz, timings were more diverse, with a primary or secondary inclination towards 135° (Fig. 6f,i,l), except when the target was 0° (Fig. 6c).

In Fig. 6m–o, where histograms are plotted separately for each FoJ, 25% of the data points each were expected to fall in the four target bins (target ± 22.5°). Allowing for slight variations in the timing, these four bins are still expected to dominate whilst a small amount of the data would fall in the neighbouring bins (target ± 67.5°). However, there is a dominance in bins of 135° ± 67.5° against the expectation. The bin encompassing 315° is the least concentrated. This suggests a general avoidance of the relative phase values in the vicinity of 315° and a notable preference towards 135° (Fig. 6m–o). The relative phase value of 135° signifies landing during upward motion at a lower platform elevation and taking off during downward motion from a higher platform elevation, while the opposite is true for 315°. Statistical analysis revealed that the number of data points that fell within the relative phase range of 135 ± 67.5° was significantly different from those in the range 315 ± 67.5° at all three FoJs. The significance level for FoJ of 2.0 Hz was $p=$ 0.024, FoJ of 2.4 Hz was $p<$ 0.01, and FoJ of 2.8 Hz $p<$ 0.01, with sufficient power, $P=$ 1.0. The aversion towards 315° increased with an increase in FoJ. Although the number of data points near the relative phase of 315° was significantly different between jumping at target frequencies of 2.0 and 2.8 Hz ($p=$ 0.05), the power was insufficient ($P=$ 0.61) to confirm this significance.

## Discussion

This experimental study aimed to capture timing and efficiency-related adaptations during rhythmic jumping on vibrating platforms. The results from additional tests involving jumping on the stationary platform serve as a benchmark. The influence of vibrations on rhythmic jumping is confirmed. Results provide novel insights into the timing-dependence of impact factor, contact ratio, mechanical work, and leg stiffness besides FoJ-dependence of leg stiffness. The key findings include participants adjusting their timing to take off from a higher platform position during its downward motion and land at a lower position during the upward motion while maintaining FoJ despite being cued. The adjustment was towards jump timings requiring lower energy input, and with appropriate contact ratio and lower forces. Thus, the jump timing behaviour relative to platform motion being dominated by the efficiency of jumping was revealed in this study.

Firstly, we establish the vibrations of 2 m/s^2^ used in this study as small amplitude within the tested frequency range of 2–2.8 Hz. This classification is justified by the greater mean jump height on the stationary platform (Fig. 3b) compared to the peak platform displacement in the scenarios with vibrations at each frequency (8.23 > 1.27 cm at 2.0 Hz, 6.15 > 0.88 cm at 2.4 Hz and 4.17 > 0.65 cm at 2.8 Hz). The effect of small amplitude vibrations on rhythmic jumping is discussed based on the experimental observations. The discussion is then extended to the potential effects of large vibrations which, due to the current lack of experimental data, should be experimentally verified in future research.

We provide evidence that rhythmic jumping is a narrow band rather than a periodic phenomenon. The variability in the kinematics and kinetics across the cycles of jumping in a trial manifests as a spread of energy around the multiples of FoJ value in the Fourier spectrum. For example, on the stationary platform, the cycle-by-cycle variability in FoJ can be observed in Fig. 2a,c and the energy spread can be seen in Fig. 2b,d. Variability is also observed for jumping at four timings on the vibrating platform (Fig. 4a–d). This finding supports those already established in the literature relating to stationary platforms^2,3,28^, and extends our knowledge to jumping on small amplitude vibrating platforms. More specifically, at 2.0 and 2.4 Hz FoJs, the percentage of jumps in the target range was similar between the stationary and vibrating platform conditions. The percentages were 77 and 78% on the stationary platform, and 79 and 79% on the vibrating platform at 2.0 and 2.4 Hz FoJs, respectively. Interestingly, the percentage of jumps within the target range of 72% was observed at the highest tested FoJ of 2.8 Hz on the vibrating platform, which is higher than on the stationary platform, where 65% were in the target range. These findings suggest an influence of small amplitude vibrations, possibly helping the participants maintain the target FoJ, especially at higher FoJ, where maintaining the target was challenging on the stationary platform. The shift to the timing of 135° could be a reason for this improved target FoJ achievement at the higher FoJ.

Additionally, the target FoJ realisation of 78% at 2.4 Hz greater than that for the other two FoJs on the stationary platform indicates that a frequency value close to 2.4 Hz is the preferred FoJ, which again is in line with the frequency choice in the literature^4,8,21,23^. Providing new insights into the consequence of the FoJ preference, our results show a greater work and impact factor near FoJ of 2.4 Hz on the stationary platform (Fig. 3c,d). This trend is not observed for jumping on the vibrating platform (Fig. 5g–i,a–c) possibly due to the influence of vibrations. A potential reason for the FoJ preference could be the natural frequency of the human body coinciding with the FoJ when jumping around 2.4 Hz. There is evidence in the literature for greater forces generated by bobbing activity at the natural frequency of the human body than other frequencies^29^. However, a confirmation specifically for jumping is needed in a future study. The literature^4^ also shows a negative correlation between the contact ratio and FoJ. However, in this study, its value at 2.4 Hz is lower than that at 2.8 Hz (Fig. 3e). The reduced jump height would have compensated for the contact ratio at 2.8 Hz.

The results also provide important insights into human adaptations towards efficiency. We provide evidence that leg stiffness is modulated in relation to FoJ. For example, on the stationary platform, there is a positive correlation between cycle-by-cycle stiffness and FoJ within the tested FoJ range (Fig. 3a) agreeing with the literature^4,12,13^ which states that humans adjust their leg stiffness to accommodate the variations in mechanical demands due to changes in the FoJ. A similar trend was also observed on the vibrating platform within the tested FoJ range (Fig. 5j–o), where leg stiffness increased with an increase in FoJ in line with the literature^9^. This is likely due to FoJ-based stiffness adjustment to reduce energy input. The faster generation of forces at higher FoJs was likely compensated by an increase in leg stiffness.

**Fig. 7:**
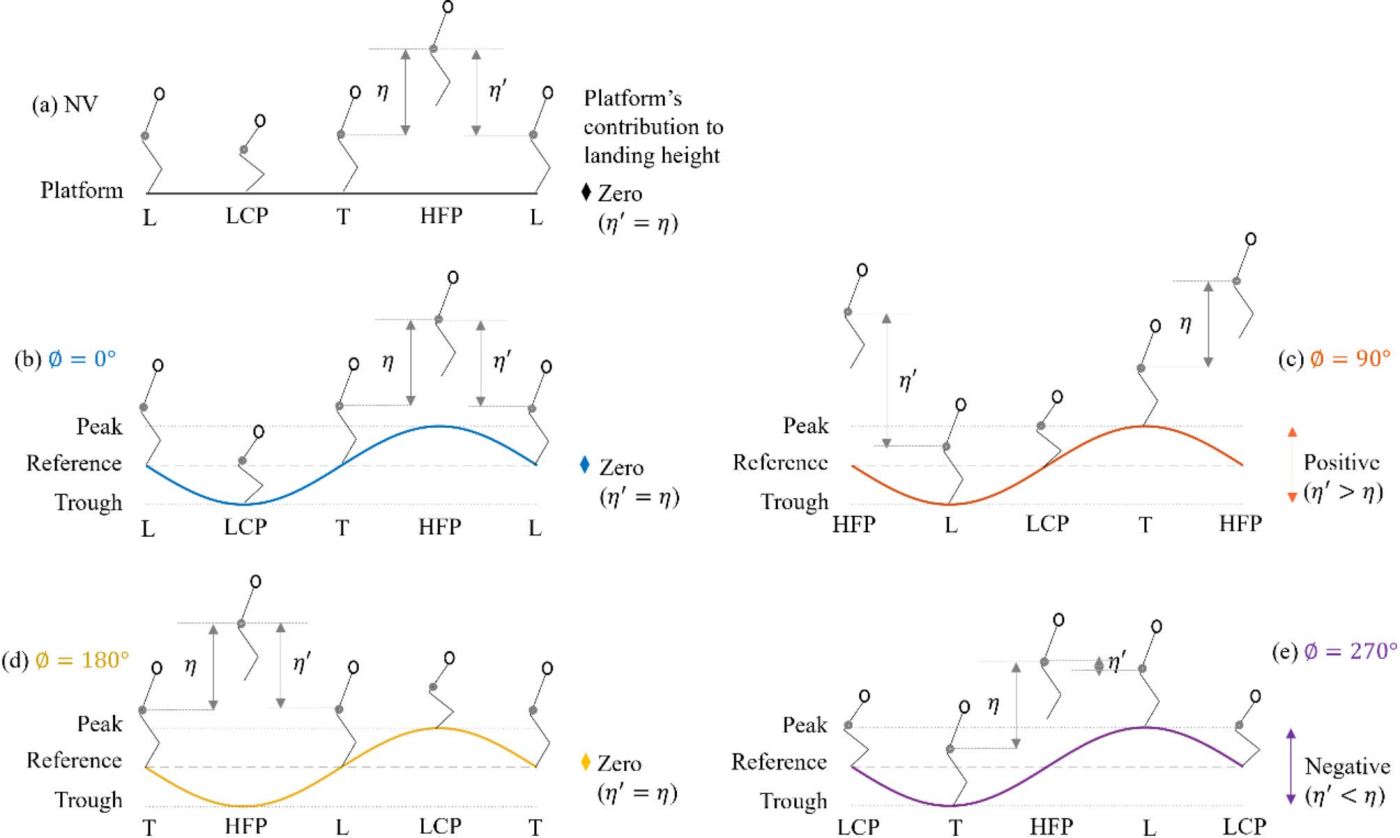
Illustration (not to scale) of (**a**) jumping on the benchmark stationary platform, and the effect of small vibrations on jumping at four timings with relative phase values: (**b**) $\Phi = 0^\circ$, (**c**) $\Phi = 90^\circ$, (**d**) $\Phi = 180^\circ$, and (**e**) $\Phi = 270^\circ$ on the vibrating platform. L: Landing, LCP: CoM’s lowest position in the contact phase, T: Take-off, HFP: CoM’s highest position in the flight phase. Body CoM, based on which the heights are calculated, is indicated using a grey circle. Jump height, $\eta$ is defined as the vertical displacement of the body’s CoM between the HFP and the take-off events in a cycle of jumping. Landing height, $\eta {\prime}$, is the vertical displacement of the body’s CoM between the landing and the HFP events, which could be different from the jump height. Jump height is assumed to represent the height attained solely due to the jumper’s effort whereas landing height depicts the combined effect of the jumper’s effort and the platform’s contribution. It is the landing height that is directly related to the generated GRFs.

Another key adaptation towards mechanical efficiency observed on the vibrating platform is related to jump timing. There is evidence for a tendency to take off from a higher platform position during the downward motion of the platform and land at a lower platform position during the upward platform motion, i.e., at relative phase values close to 135°. To explain this efficiency-dependent behaviour relating to jump timing, Fig. 7 provides a physical illustration of human and platform kinematics while jumping at four timings (Fig. 7b–e) on a platform vibrating at a small amplitude in comparison with jumping on the stationary platform (Fig. 7a). When jumping on the stationary platform, as seen in Fig. 7a, the jump height is equal to the landing height. However, on the vibrating platform, the landing and take-off positions can potentially be influenced by the platform’s interference, resulting in a landing height different from the jump height (Fig. 7c,e). Nevertheless, the jump height on the vibrating platform for different jump timings (Fig. 7b–e) is likely to remain the same as that on the stationary platform (Fig. 7a) for the same effort produced by the jumper.

Figure 7b illustrates jumping at 0° relative phase with the platform. In this scenario, the jumper takes off from the platform’s reference position and lands at the same location after completing the flight phase. This results in a landing height equivalent to the jump height, much like the experience of jumping on a stationary platform. Consequently, the impact factor, contact ratio, work and leg stiffness closely resemble those observed in stationary conditions (Fig. 5). It is hypothesised that this equivalence holds as long as the platform’s displacement remains smaller than the jump height. However, if the vibrations become substantial, the jumper must increase their jump height to sustain rhythmic jumping. In some cases, the platform’s displacement may exceed the physiological limits of jump height, making rhythmic jumping unsustainable as the platform’s movements interfere with the jumper’s motion during the flight phase. This is most likely the rationale for the previous experimental findings^5,25^, emphasising the impossibility of jumping at the vibration frequency and the reduction in forces when exposed to substantial vibrations. The maximum platform displacement observed in those tests for a platform vibrating at 2.0 Hz was an extraordinary 13.5 cm^5^. This value is much greater than the peak platform displacement of 1.27 cm corresponding to 2 Hz vibration and the mean jump height of 8.23 cm for 2.0 Hz jumping in this study. Therefore, the physical interpretation along with the experimental results presented in this study provides the rationale for the possibility of rhythmic jumping at the frequency of small amplitude vibrations. Jumping at the vibration frequency can lead to resonance and affect the vibration serviceability of structures.

Jumping at 90° relative phase entails taking off from the platform’s peak position and landing in the platform’s trough (Fig. 7c). Therefore, the landing height is greater than the jump height due to the downward motion of the platform while the jumper is in flight. There is evidence that the jumper lands after the beat when the 90° relative phase was targeted, which can cause a shift in the timing towards 135° (Fig. 6e,f). This is because of a longer flight duration due to greater landing height and, subsequently, a reduction in the contact ratio (Fig. 5d–f). Additionally, the jumper might not prefer a lower contact ratio due to the higher cost of faster muscular force generation^4^, therefore avoiding 90°. The increased landing height also results in an increased impact factor (Fig. 5a–c). The net mechanical work (Fig. 5g–i) and leg stiffness (Fig. 5j–o) are greater than those for the rest of the timings. The energy stored during compression is greater than the energy released during extension and the legs are stiffer during extension than compression (Fig. 4c). Importantly, it is crucial to note that the platform does not interfere with the jumper’s flight phase due to its downward motion (Fig. 7c). However, vibration amplitudes higher than those used in the tests could amplify the platform’s contribution to the landing height, which could potentially increase the risk of injury due to large contact forces. Therefore, sustaining rhythmic jumping at this timing is challenging and the jumpers are likely to avoid it when vibrations are not small.

Jumping at 180° relative phase, illustrated in Fig. 7d, is the opposite of the 0° relative phase. The landing and take-off positions remain the same although the direction of platform motion is the opposite. The platform moves to its trough position and returns to the reference position as the jumper completes the flight phase. This results in a similar landing height, impact factor, contact ratio, work and leg stiffness as jumping on the stationary platform (Fig. 5). If the vibrations are large, unlike the timing of 0°, the platform motion does not affect the jumper, as the platform is below its reference position throughout the jumper’s flight phase and hence does not interfere with jumping. Therefore, there is a possibility of sustaining rhythmic jumping at this timing even when vibrations are large. However, this needs to be verified experimentally in the future.

Jumping at the 270° relative phase, as shown in Fig. 7e, is the opposite of the 90° relative phase. The jumper takes off from the platform’s trough and lands at the peak. The platform moves upward during the flight reducing the landing height. Legs are stiffer during compression than extension, resulting in a net negative work (Fig. 4a). When vibrations are not small, the jumper has to raise their CoM even higher to avoid premature contact and sustain flight. Raising CoM to overcome the platform’s negative contribution to landing height demands additional effort from the jumper. This situation is akin to jumping on a pile of sand where energy is dissipated^22,23^. The legs act like work-producing actuators^22,23^. The jumper lands ahead of the beat (Fig. 6j–l), as the situation is unsustainable. Consequently, the contact duration increases (Fig. 5d–f), and the impact factor decreases (Fig. 5a–c), in agreement with the force reduction observed in the literature^5,25^. Depending on how long the jumper can sustain flight, the attained relative phase can vary between 90° and 270° (Fig. 6j–l). Jumping at the relative phase of 270° is attainable only at low vibration levels and at low FoJs. For higher FoJs and large vibrations, maintaining this timing becomes extremely challenging, so jumpers avoid it.

Results show that relative phase values close to 135° were most achieved (Fig. 6m–o) rather than the four cued timings. Jumping at the 135° relative phase is the most advantageous timing that involves taking off from a higher platform position during the downward motion of the platform and landing at a lower platform position during the upward platform motion. This choice likely ensures that the platform does not disrupt the jumper’s flight, the contact ratio is sufficient, injury risks are avoided, positive work is produced, and additional energy input is not required. This situation is similar to jumping on an elastic surface where legs act like energy-conserving springs^22^. The choice of jumping close to the timing of 135° and avoiding 315° is confirmed as statistically significant in this study.

The occurrences of jumping close to the timing of 135° and avoiding 315° increased with an increase in FoJ (Fig. 6). At the lowest FoJ of 2.0 Hz tested in this study, some participants took off a quarter cycle before the beat in some trials (Figs. 4 and 6). They took off before the beat likely due to their inability to wait for the metronome beat due to slow jumping at 2.0 Hz^2,5^. However, the dependence of timing choice on the FoJ could not be confirmed due to a lack of sufficient statistical power owing to large variability.

In summary, this study provides evidence for the sustainability of rhythmic jumping at the frequency of small amplitude vibrations. Vibrations affect jumping by altering the landing height depending on the jump timing, referred to as the mechanical interaction between the human and platform kinematics. Apart from this mechanical interaction, there are also behavioural effects. Humans choose their jump timing relative to vibrations and adjust leg stiffness to maximise the efficiency of jumping on vibrating platforms just like they choose FoJ and adjust stiffness on stationary platforms. This study detailed the energy flow, contact ratio and impact factor associated with the timing of jumps relative to vibrations and provided evidence for jump timing being dominated by the efficiency of jumping. At the preferred timing, the platform does not disrupt the jumper’s flight, positive work is produced, the mechanical cost is low, the contact ratio is sufficient, and injury risks due to large contact forces are low. Jumping at the preferred timing on the vibrating platform results in generated forces and net positive work greater than those on the stationary platform, thus significant for human-induced load calculation on lively assembly structures. This knowledge will help in improving the understanding and modelling of human-structure interaction to help reduce conservativism, uncertainty, and risk in structural design and as a means of optimising the amount of construction material. Future research should consider jumping at a frequency different from the vibration frequency.

## Methods

### Test protocol

The test protocol was approved by the Research Ethics and Governance team of the University of Exeter. All methods were performed in accordance with the relevant guidelines and regulations set out by the World Medical Association Declaration of Helsinki^30^. Six male and four female healthy participants (age 25.50 ± 4.09 years, body mass 62.96 ± 7.45 kg, and height 169.70 ± 6.09 cm) provided written informed consent and participated in the study. Inclusion criteria were that participants be over 18 years of age, healthy and free of musculoskeletal injury, not prone to motion sickness/vibration sensitivity, and not suffering from locomotion impairments. Additionally, pregnancy was an exclusion criterion for the female participants.

Tests were conducted in the VSimulators facility^31^ at the University of Exeter during multiple visits. Three test configurations were run on the stationary platform (3 FoJs), and twelve on the vibrating platform (4 timings × 3 FoJs), with three repeats of each configuration. On the stationary platform, participants were cued with a metronome to jump at 2.0, 2.4 and 2.8 Hz for 40 jumps. On the vibrating platform, for which the sinusoidal vertical vibration of the platform had an amplitude of 2 m/s^2^, participants were asked to jump in time with a metronome at frequencies of 2.0, 2.4 and 2.8 Hz, while the platform also oscillated at the matching frequency. Specifically, on the vibrating platform, the metronome was set to beat at four timings relative to the platform’s displacement, cueing the participant to land at four platform positions: the reference position and on the way down or mid-down (A), lowest position or trough (B), reference position and on the way up or mid-up (C), and highest position or peak (D). The participants were instructed to perform in-place bilateral rhythmic jumping and coincide their landing with the metronome beat. No restrictions were set for arm swing.

The test design was counterbalanced by employing randomisation of test configurations, which refers to the order in which the participants were exposed to the test configurations^32^. This approach was used to minimise the potential effects of motor learning. Additionally, the order of test configurations for the three repeats varied independently from one another, as they were randomised separately. The data from 15 test configurations utilised in this study are part of a larger experimental campaign that involved 39 test configurations per participant. The remaining 24 configurations, not studied here, had a metronome frequency different from the respective vibration frequency.

The duration of a trial was determined so to ensure the recording of 40 cycles of jumping. The data acquisition lasted 20 s for jumping at 2.0 Hz, 17 s for jumping at 2.4 Hz, and 15 s for jumping at 2.8 Hz. These durations of the trials were chosen to both not exhaust the participants by unnecessarily prolonging their jumping and to have enough jumping cycles to capture inherent variations in GRF ^2^. There were 20 s resting intervals between consecutive trials. In addition, after completing a session of 13 trials (the first trial in every session of 13 trials was a practice trial and was not used for analysis), the participants were provided with a minimum rest period of ten minutes, allowing them to rest for as long as necessary to minimise fatigue. In most cases, the resting breaks were extended beyond 10 min due to technical considerations, although none of the participants requested an additional break. On days with multiple participants performing tests, they took turns to complete their sessions. Before commencing each session, an assessment was made to ensure that the participant was not fatigued. This involved observation and direct communication with the participant. If any participant showed signs of fatigue, the tests were halted for that participant and resumed on another suitable day. There was one such instance.

Kinetic and kinematic data for individuals jumping were collected during all trials. The VSimulators is a multi-axis shake table equipped with nine 1.2 m × 1.2 m AMTI BP12001200 force plates^33^, embedded in the floor to measure the GRF at a sampling rate of 1000 Hz. An OptiTrack motion capture system^34^, operating with 16 OptiTrack Prime 13 cameras, was used to track trajectories of reflective markers attached to the human anatomical landmarks (100 Hz). The conventional full-body marker set comprising 39 markers was adopted^34^. On the day of the tests, the participants wore a motion capture suit to which the reflective markers were attached. Additionally, four markers were placed at the four corners of the platform to enable the tracking of platform displacement.

### Data pre-processing

To analyse FoJ, jump timing, impact factor, jump height, contact ratio, mechanical work done and leg stiffness, the vertical GRFs and the vertical displacements of the body CoM and the platform were utilised. Spline interpolation was performed to fill the missing marker data. GRF and marker position data were low-pass filtered using a fourth-order zero-phase Butterworth filter with a cut-off frequency of 10 Hz. The measured force signal included the inertia of the force plate on which the participant performed jumping. This inertia force was subtracted from the signal before the analysis. To obtain the time history of CoM displacement, trajectories of 23 out of 39 markers placed on the head, shoulders, seventh cervical vertebrae, elbows, wrists, fingers, hips, knees, ankles, heels, and toes were utilised. The human body was divided into 14 rigid segments to represent the head, trunk, upper arms, forearms, hands, thighs, shanks, and feet. Inertial parameters, including mass and CoM position, of each segment were derived using the anthropometric model described by de Lava^35^. Subsequently, a weighted average calculation was applied, considering the inertial parameters of each segment to determine the position of the body CoM^35^. Furthermore, the displacement of the CoM relative to the platform was computed. The static component from the CoM and platform displacements were further removed so that their mean displacement values were zero. The final 25 cycles of the data recorded in each trial were selected for analysis. The CoM displacements and normalised GRFs were also visualised in the frequency domain by plotting their Amplitude Spectral Density (ASD) employing the Fast Fourier Transform function in MATLAB^36^.

### Parameter extraction

The FoJ, impact factor and contact ratio achieved by the participants in each trial were extracted on a cycle-by-cycle basis from the GRF time history as per Abraham et al.^24^. The FoJ was calculated as the inverse of the time difference between consecutive landing events. The impact factor was obtained as the peak GRF normalised with the participant’s body weight for each cycle. The contact ratio was found as the duration for which the participant exerted more than 10% of their body weight in each cycle^17^. Furthermore, jump height was obtained as the peak CoM displacement from the mean position on a cycle-by-cycle basis. Mechanical work was quantified as the area under the force–displacement graph as per Czaplewski et al.^27^ and leg stiffness was calculated as the mean slope of the graph based on White et al.^9^.

The cycle-by-cycle jump timings relative to vibrations were computed as the relative phase between the displacements of CoM and the platform based on coordination patterns between them. Vector coding techniques were employed to obtain the coordination^37,38^, i.e., whether the platform and body CoM were moving in the same direction (in-phase coordination) or opposite direction (antiphase coordination). CoM displacement during both compression and extension phases showing in-phase coordination with platform motion will result in a relative phase of 0°. Conversely, CoM motion during compression and extension phases in antiphase coordination with platform motion will yield a 180° relative phase. Antiphase coordination during compression and in-phase coordination during extension will result in a 90° relative phase. In-phase coordination during compression and antiphase coordination during extension will give a 270° relative phase. Based on these four cases, the relative phase ($\Phi$) can be quantified as angular values in the range [0, 360°) from the fraction of points with in-phase coordination during compression (${IP}_{ext}$) and extension (${IP}_{comp}$) phases as^39^:

1 $$\Phi = a\tan 2d\left( {IP_{ext} - 0.5,IP_{comp} - 0.5} \right) - 45^\circ$$

**Fig. 8:**
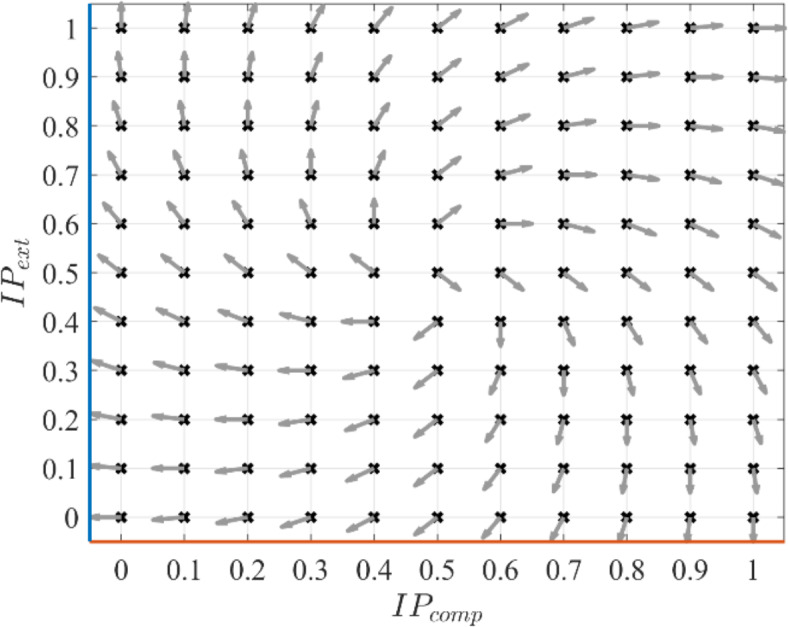
Illustration of relative phase values as indicated by the angle made by the arrows with the positive horizontal axis ($x$-axis) corresponding to different coordination patterns based on the duration of in-phase coordination during the extension and compression of the contact phase.

The inverse tangent function $atan2d$ in Eq. (1) is a MATLAB function^36^ that takes two arguments, $(y,x)$, and returns the angle between the positive $x$-axis and the point $(x,y)$ in degrees. It allows for the calculation of angles in all four quadrants of the Cartesian coordinate system. Figure 8 graphically represents Eq. (1). All data points in the displacement time histories of CoM and platform corresponding to the lower half of each cycle of jumping (comprising most of the contact phase) were used for relative phase calculation. This method overcomes the limitations of discrete methods that rely on the occurrences of specific events^24,26,40^ and complex continuous methods^41,42^ for quantifying the relative phase.

### Statistical test

A one-way Repeated Measures Analysis of Variance (RM ANOVA) test was conducted (SPSS, version 29.0.2.0 (20)^43^), with a single factor, the target FoJ. The dependent variables analysed were the percentage of cycles with a relative phase value falling in each bin of the histogram showing the distribution of the relative phase (Fig. 6). Significance was set a-priory as $p\le$ 0.05^44^. To account for multiple comparisons, post-hoc pairwise comparisons were performed with Bonferroni adjustment. To assess the adequacy of the sample size, power calculations were performed for each test. A test with a power value ($P$) below 0.8 is generally considered underpowered, indicating insufficient statistical power to detect meaningful effects^44^. Insufficient statistical power can compromise the ability to detect meaningful effects and draw definitive conclusions. Power becomes insufficient when the variability is large and can be improved with an increase in sample size.

## Data Availability

The dataset used and/or analysed during the current study is available from the corresponding author upon reasonable request.
